# Lottery incentives for smoking cessation at the workplace: design and protocol of the smoke-free lottery - a cluster randomized trial

**DOI:** 10.1186/s12889-022-14915-x

**Published:** 2023-01-11

**Authors:** Koen van der Swaluw, Marieke Hiemstra, Mattijs Lambooij, Eline Roordink, Nina van der Vliet, Else Zantinge, Karin Proper, Marcel Zeelenberg, Henriette M. Prast

**Affiliations:** 1 National Institute of Public Health and the Environment (RIVM), Centre for Nutrition, Prevention and Health Services, 3720 BA Bilthoven, The Netherlands; 2 Department of Economics and Business Economics, Nijmegen School of Management, Radboud University, 6500 HK Nijmegen, The Netherlands; 3 National Institute for Public Health and the Environment (RIVM), Centre for Sustainability, Environment and Health, 3720 BA Bilthoven, The Netherlands; 4 Tilburg University Graduate School, Tilburg School of Social and Behavioral Sciences, 5000 LE Tilburg, The Netherlands; 5 National Institute of Public Health and the Environment (RIVM), Centre for Health and Society, 3720 BA Bilthoven, The Netherlands; 6 Amsterdam UMC, Department of Public and Occupational Health, Amsterdam Public Health Research Institute, Amsterdam, The Netherlands; 7 Tilburg University, Department of Social Psychology, Tilburg School of Social and Behavioral Sciences, 5000 LE Tilburg, the Netherlands; 8 VU Amsterdam, Department of Marketing, School of Business and Economics, De Boelelaan 1105, 1081 HV Amsterdam, The Netherlands; 9 Tilburg University, 5000 LE Tilburg, the Netherlands; 10 Dutch Senate, 2500 EA Den Haag, The Netherlands

**Keywords:** Smoking cessation, Workplace, Incentives, Commitment device, Lottery, Deadlines, Behavioral economics

## Abstract

**Background:**

Smoking is the leading behavioral risk factor for the loss of healthy life years. Many smokers want to quit, but have trouble doing so. Financial incentives in workplace settings have shown promising results in supporting smokers and their design influences their impact. Lotteries that leverage behavioral economic insights might improve the effectiveness of workplace cessation support.

**Methods and design:**

We examine in a cluster randomized trial if a workplace cessation group training paired with lottery deadlines will increase continuous abstinence rates over and above the cessation training alone. Organizations are randomized to either the control arm or lottery arm. The lotteries capitalize regret aversion by always informing winners at the deadline, but withholding prizes if they smoked. In the lottery-arm, winners are drawn out of all participants within a training group, regardless of their smoking status. In weeks 1-13 there are weekly lotteries. Winners are informed about their prize (€50), but can only claim it if they did not smoke that week, validated biochemically. After 26 weeks, there is a long-term lottery where the winners are informed about their prize (vacation voucher worth €400), but can only claim it if they were abstinent between weeks 13 and 26. The primary outcome is continuous abstinence 52 weeks after the quit date.

**Discussion:**

There is a quest for incentives to support smoking cessation that are considered fair, affordable and effective across different socioeconomic groups. Previous use of behavioral economics in the design of lotteries have shown promising results in changing health behavior. This cluster randomized trial aims to demonstrate if these lotteries are also effective for supporting smoking cessation. Therefore the study design and protocol are described in detail in this paper. Findings might contribute to the application and development of effective cessation support at the workplace.

**Trial registration:**

Netherlands Trial Register Identifier: NL8463. Date of registration: 17-03-2020.

**Supplementary Information:**

The online version contains supplementary material available at 10.1186/s12889-022-14915-x.

## Background

Tobacco use is causing 8.7 million deaths every year worldwide and remains the largest behavioral risk factor for noncommunicable disease and loss of healthy life years [1]. Moreover, approximately one third of socioeconomic inequalities in mortality are attributable to smoking [2]. Stopping smoking is, therefore, one of the most direct routes to increasing life expectancy up to 10 years and reducing socioeconomic inequalities in health [2, 3].

Next to the health burden, tobacco use also imposes substantial economic costs via health expenditures and through productivity losses [4]. Smoking is associated with reduced work performance and a 31% higher likelihood of workplace absenteeism; smokers annually take approximately three more sick days than non-smoking colleagues [5–7]. Estimates show that employers suffer an excess expense of $5816 annually to employ a smoker in comparison to a non-smoker [7].

Therefore, employers might benefit from facilitating smoking cessation support at the workplace. There is strong evidence that workplace cessation interventions such as group counseling, are effective in helping people to stop smoking [8]. In addition, especially among disadvantaged populations, accessibility, proximity and financial compensation are important aspects in the uptake of cessation interventions [9, 10], whereas broader and untargeted interventions may be less effective [9, 11]. Smoking cessation support at the workplace has the benefit of being a suitable nearby and familiar intervention-context, which can aid the necessary improvements in behavioral intervention design that are needed to realize more equal smoking cessation outcomes across socioeconomic positions [12].

Although the majority of smokers do not want to smoke and often try to quit, most attempts fail [13]. There are multiple factors that make quitting challenging. In addition to overcoming a physical addiction, quitting is difficult due to the time-gap between the immediate effort that is needed to quit and the mostly long-term benefits of quitting [14, 15]. In a ‘cold’ deliberative state smokers can genuinely set a long-term health goal, but when they are in a more ‘hot’ affective state, they may succumb to (overly present) immediate temptations. Tobacco is widely available and the nearby (social) benefits of smoking then outweigh the delayed benefits of quitting, resulting in lower levels of goal attainment [16, 17].

Financial incentives have the potential to help overcome this pattern because of their more immediate nature [18, 19]. Literature reviews show there is high-certainty evidence that incentives improve smoking cessation rates across mixed populations [20]. For example, a randomized trial with 61 businesses finds that an individual €350 incentive on top of a workplace cessation group training increased the proportion of abstinent employees 1 year later with 15 percentage points, compared to an only group training treatment [21]. Likewise, higher rates of smoking cessation up to 18 months are found when employees were offered a financial incentive ($750) on top of a smoking cessation program [22].

The configuration of incentives can influence their effect on behavior [23, 24]. Researchers crafting incentives for smoking cessation have to make decisions about the incentive’s form, timing, frequency, certainty and magnitude (see Adams et al., for a framework [23]). Literature reviews show no clear association between quit-rates and incentive size [20, 25]. While this largely shows a gap in research, it might also indicate that other important design features influence the effect of incentives on behavior [26].

A promising design used by behavioral economists and medical professionals is the use of *regret lotteries*, which have been demonstrated to support weight loss [27], medication adherence [28] and physical activity [29, 30] at relatively low costs [31] across differing populations [32]. By their design, the lotteries aim to tap into multiple psychological insights on decision-making, with the goal to gain as much health out of every euro spent. Most prominently, all participants can win the prize at the deadline and the winner is always informed about the outcome. However, winners can only keep their prize if they attained their own prespecified health goal. The promise of this counterfactual feedback is meant to leverage anticipated regret [33]. Although these lotteries have been effective in supporting multiple health behaviors with an intertemporal character, to the best of our knowledge, this particular design remains untested for smoking cessation.

### Aim and hypothesis

The aim of this paper is to describe the protocol for a cluster randomized trial “The Smoke-Free Lottery” which aims to investigate whether lotteries will increase the effectiveness of tobacco-cessation group training at the workplace. It is hypothesized that the lotteries increase continuous abstinence rates over and above cessation training alone.

## Methods/design

### Setting

This trial takes place in the Netherlands. Currently, 15 % of the adult Dutch population smokes daily (~ 2.1 million), which is lower than the average in the European Union. The percentage of daily smokers in the lowest income group is about twice as high as that in the highest income group. Likewise, lower educated adults include a double to triple percentage of smokers in comparison to the higher educated [34].

Approximately 31% of smokers took a serious attempt to quit in 2021, which is significantly lower than in 2020 (36%) [34]. Health insurance is mandatory and fully covers a registered cessation training (maximum of 1 per year). Medication during cessation training is covered by most insurers and the training itself is exempt from deductibles.

### Study design

**Fig. 1:**
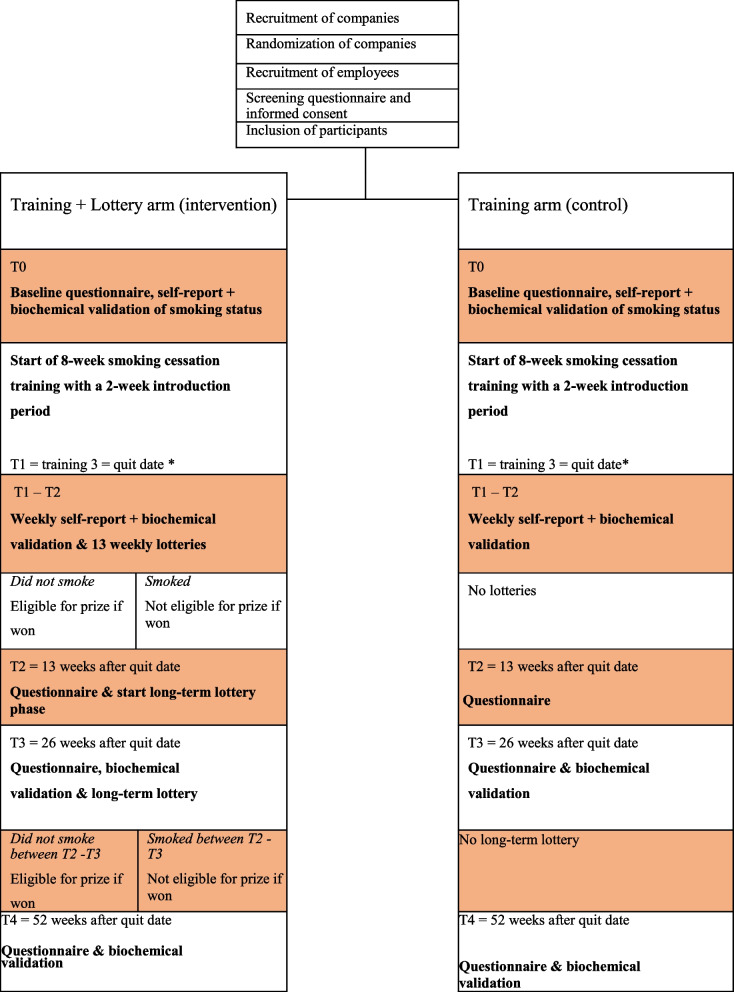
Trial flow and event schedule

We propose a two-arm, parallel group, cluster-randomized trial running for 52 weeks in 16 organizations (clusters) across the Netherlands. We aim to assign organizations to receive either a smoking-cessation training (8 companies) or a smoking-cessation training plus the smoke-free lottery (8 companies). Participants in both arms participate in an identical 8-week smoking cessation group training at the workplace. Participants in the lottery condition will additionally participate in 13 weekly lotteries starting from the prespecified joint quit date, complemented with a long-term lottery after 26 weeks. A schematic representation of the trial is presented in Fig. 1. The trial protocol and materials were reviewed and approved by the Radboud University Ethical Review Board (ECSW-2019-114). The study is registered in the Dutch Trial Register (NTR NL8463) and lottery drawings are performed by an independent notary.

## Participants

### Recruitment

For this trial we cooperate with SineFuma, a Dutch company that delivers smoking cessation training at the workplace. By email, flyers, newsletters, network contacts and social media, we will recruit organizations over the course of 1.5 years to facilitate a group cessation training and participate in the study. SineFuma also informs their new clients about the possibility to join the present study.

The recruitment of companies follows several steps, designed minimize recruitment bias at the company level and maximize clarity and ease for the companies. As a first step, companies are informed about the study and the fact that they will be randomized if they participate. A study staff member checks with the company if they meet all eligibility criteria, verbally or written. If a company agrees with randomization and meets the criteria, the third step is that the company sets up the planning for the group training with SineFuma and signs their written offer. Hereby the company commits to organizing and financing the training, but they do not know their allocation yet. For the next step, only after SineFuma communicates to the study staff that the interested company has financially and logistically committed to organizing the group-training, do we start the randomization procedure. This makes that companies can decline to participate in our trial because they dislike the idea of randomization, but minimizes the risk of recruitment bias by companies cancelling the entire program because they disagree with their allocation.

For the recruitment of employees, SineFuma typically organizes an information meeting for their cessation training at the workplace. The training and meeting are advertised internally by the companies via email, intranet, flyers and posters. At the information meeting, the study staff additionally informs the smokers about the study, using our folder, video and slides and explains how employees can enroll. Employees are informed that they can also join the group training without enrolling to the study.

Employees interested in the study can submit their email address. An information letter and screening questionnaire are sent by the study staff. Candidates can choose to participate in the study until the start of the first session of the smoking cessation program. This will be at least 1 week, up to several weeks, depending on the planning of the training.

Because we start recruitment of employees after allocation of the cluster, the lotteries might attract a proportion of participants that otherwise would not have joined the 8-week long cessation program. As such, the control group might to some degree exist of higher cessation-committed participants, reducing a potential treatment effect. To prevent this recruitment bias as much as possible, we designed three securities in our recruitment. First, we use highly similar standardized recruitment materials informing about a) the value and meaning of participation in a scientific study and b) the advantages and disadvantages of the required measurements and surveys. Second, participants are informed about the treatment that is relevant to them and that evaluating this treatment is the goal of our study. This means that we do not tell participants that they were randomized, therefore missed out on receiving incentives and are next being compared to the other treatment. Third, recruitment within the companies is focused largely on the cessation training through communication materials by SineFuma, as this is the core of the commitment. Candidates are informed about the study generally after information about the 8-week training.

### Eligibility criteria

For organizations to participate, the management should be willing to pay for the training and, if assigned to the lottery arm, to pay for the lotteries. They have to agree to participate prior to randomization. The management allows their employees to participate in the group training sessions and carbon monoxide (CO)-measurements during or shortly before or after working hours on a location hosted by the employer. After the outbreak of Sars-Cov-2, the training is hosted mostly online (see below), which dismisses the location-criterion.

For employees to participate, they need to have smoked tobacco (no e-cigarettes) for at least one pack-year (= number of daily packs x years), smoke daily, are willing to quit, want to join the group training and are at least 18 years old. Employees that are not able to read or speak Dutch are excluded from participation in this study, because the cessation training is in Dutch.

After expressing interest in the study, participants receive an information letter, a screening questionnaire and a written informed consent form. Ineligible candidates receive an e-mail and can participate in the cessation training, but not in the study and thus the lotteries.

### Randomization

Organizations (clusters) are randomized using a computer-generated biased urn schedule prior to the recruitment of individual participants. The biased urn method entails that the allocation probability changes based on the current balance [35]. As such, the probability of allocation to either arm depends on the level of organizations already randomized to that arm. Larger organizations with distinct subsidiaries or autonomous sub-departments can be randomized separately, only if treatment contamination can be avoided.

Allocation concealment at the organization level is ensured by first including the organization and randomizing after. Only after an explicit commitment by the organization, randomization is requested by the recruiting staff member to the randomizing staff member, in order to conceal knowledge of the upcoming assignment by the recruiters. We decided not to randomize at the participant level to avoid disappointment and possible attrition and to ease and unify recruitment of employees by employers.

### Blinding

Participants are not informed about the treatment in the other arm. Nonetheless, blinding cannot be fully guaranteed as a result of how organizations are publicly recruited. We do not inform participants that their treatment will be compared to another treatment. Researchers and the management of the companies could not be blinded due to the nature and multi-party coordination of the trial.

### Sample size calculation

In a meta-analysis, Haff et al. [32] analyzed lottery trials targeted at various health behaviors, with a pooled success percentage of 57.5% in the lottery condition versus 22.6% in the control condition. A sample size calculation for detecting a 0.35 difference between proportions at *p* < .05 and a power (1-*β*) of 90%, indicated a sample size of 40 per condition. With an intra-class correlation coefficient of .05, based on a CRT by Van den Brand and colleagues [21] and an estimated cluster size *m* of 8, a design effect (1 + (*m*-1) x ICC) of 1.35 an effective sample size of 54 per condition was estimated. We aim to recruit a minimum of 64 participants per condition (meaning 8 clusters per condition), allowing 15% participant attrition over time. Based on the power calculation, we aim to assign 16 clusters to receive either a smoking-cessation training or a smoking-cessation training plus the smoke-free lottery.

### Retention

As a compensation for their participation in the study, participants are promised and given €30 at the end of the study, regardless of smoking status. To further prevent attrition and increase commitment to the study, we will hand all participants gadgets with the institute’s logo throughout the study (a water flask and mug) and send them a birthday and Christmas card on behalf of the study staff. Participants who do not respond to surveys or other measurements, receive a reminder text message and email.

## Intervention

This trial compares a control-arm to an intervention-arm. All participants get an anonymized trial-ID at the start of the trial.

### Control arm

As standard treatment, participants join the evidence-based group cessation training organized by SineFuma, which has been developed independent of this study and is based on Withdrawal-oriented Therapy [36] and Motivational Interviewing [37]. With certified trainers, SineFuma provides group training at work throughout the Netherlands. Among other topics, smokers learn to cope with cravings, social pressure and physical difficulties such as weight gain. The pre-existing training capitalizes peer support and informs smokers about the possibility to use medication to aid their cessation attempt. The group training has 7 meetings of 90 minutes, spread over 8 weeks and take place online or at the workplace. After 2 weeks of preparation and group forming, participants get a pause-week and jointly quit in the third meeting (in week 4). In the fourth meeting, participants should be smoke-free for 1 week. Participants are handed a personal CO-meter, that is linked to their smart-phone (see *measures*). Typically, groups can consist of a maximum of 16 participants, with an average of 10 [21]. After the outbreak of SARS-CoV-2, Sinefuma offers the training as e-health training online in identical form with a maximum group size of 8 participants.

### Intervention arm: smoke-free lottery

#### Weeks 1 to 13

**Fig. 2:**
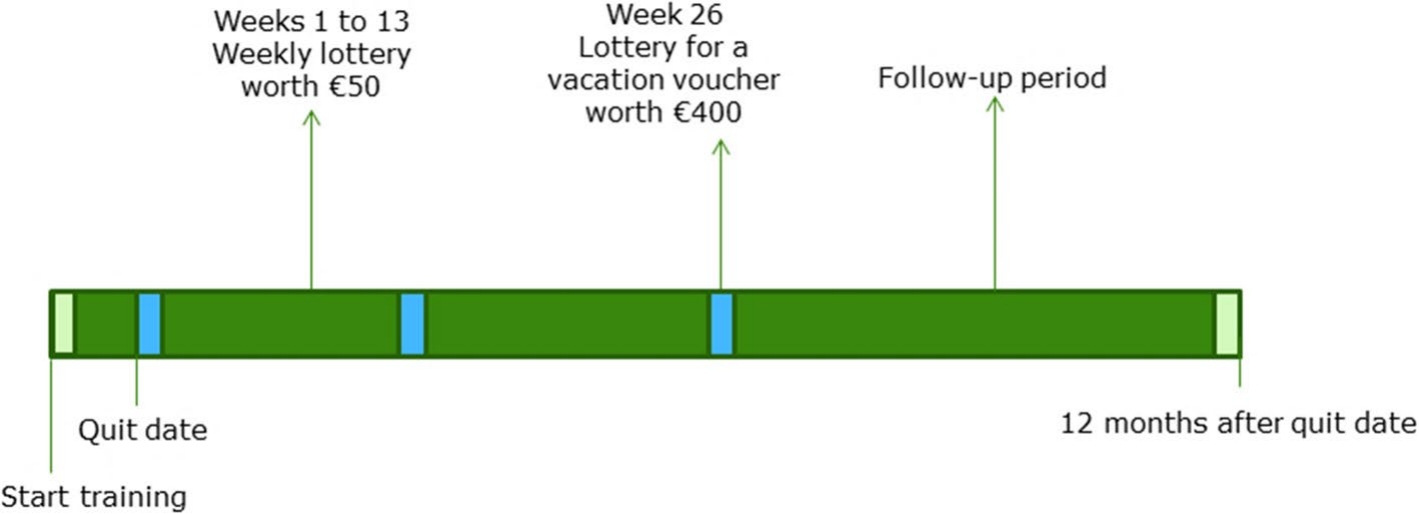
Timeline of the Lotteries

Participants in the lottery arm receive the same treatment as the control arm in all aspects and additionally participate in lotteries. Prize sizes were chosen such that the weekly expenses per participant in proportion to the Dutch minimum wage were similar (0.6%) to a previous effective instance of this lottery [31]. We also mimic the lottery-deadline schedule in the best performing trial-arm to promote gym attendance in Van der Swaluw et al. [31]. Previous research shows that most relapse to smoking occurs in the early stages after quitting [38, 39], and that the added value of intervention is achieved mostly in the first 3 months [40, 41]. As a result, is has been proposed that cessation interventions should be ‘front-loaded’ [38]. Accordingly, we offer repeated weekly short-term lottery deadlines, immediately after the quit date. The lottery timeline is illustrated in Fig. 2.

The first lottery deadline is 1 week after the joint quitting moment in the third training. From the fourth training, lottery participants can win €50 every week for 13 weeks. The winner is drawn out of all participants within a group using the trial-ID, regardless of their smoking status. Participants only receive their prize if they did not smoke that week, as confirmed by the CO-measurement. Participants are informed by text message and email about whether they have won the prize and whether they receive their prize.

Lottery winners who need to forfeit their prize because they smoked, will learn about their forgone prize. All other participants will be informed about whether the prize is awarded or not, but not to whom. Every week offers a new chance to win, irrespective of prior performance. If the winner is not eligible for the prize, the money is forfeited. If the winner has previously withdrawn from the study, the notary will draw a new winner.

To summarize, when participants are selected as a winner, they are informed about this. Next, if they smoked in the week before the draw, they do not get the prize. Other participants are then also informed that the winner did not get the prize because he or she smoked.

#### Weeks 14-26

After 26 weeks, all participants can win a family vacation voucher (worth €400). Again, the winners are drawn out of all participants within a group, regardless of their smoking status. The winner is always informed by text message and email. However, the winner only receives her prize if she was abstinent between weeks 14 and 26, as confirmed by the CO-measurement. If the winner is not eligible for the prize, a new winner is drawn until the prize can be awarded. All other participants will be informed that the prize is awarded or not, but not to whom.

The lotteries tested in this trial are distinct from conventional lotteries or quit and win contests because winning the lottery is not conditional on performance, but being able to claim the prize is. Participants are always in the drawing, irrespective of their performance. With this design, the lottery incentives take more the form of a commitment device [42], where people accept a presented deadline with the potential of finding out that they won a prize, but losing it because they did not stick to their own goal of not smoking.

As such, there are multiple design aspects that are meant to support people in achieving their own goal. First, the lotteries offer a vivid deadline with nearby consequences. This can help overcome an intrapersonal conflict between a farsighted *planner* that wants to quit smoking and shortsighted *doer* that wants to enjoy a cigarette. According to this model of self-control [43], people try to control their future myopic behavior to attain their long-term goals by restricting their future freedom of choice. Acceptance of a deadline with immediate feedback has been shown to achieve this [42, 44] and is therefore designed to obstruct the tendency to postpone the quitting attempt to enjoy a cigarette right now.

Second, people tend to overestimate their chances of winning a lottery [45]. Decision-making under risk is known to be influenced by emotional assessments of the outcomes and emotions at the time of the decision [46]. These emotions result in overweighting of small probabilities and especially for vivid outcomes [46]. As such, the potentially emotional effect of the lottery outcome is designed to increase the importance of the deadline.

Third, the lotteries -also dubbed *regret lotteries-* leverage the tendency to anticipate and avoid regret by only awarding prizes at the prespecified deadline to lottery winners who attained their health goals and always informing unsuccessful lottery winners what they would have won, had they attained their goal [47]. This way, participants know that they can compare the outcome of their decision to the counterfactual outcome, had they decided differently. If the alternative decision-option turned out better than the chosen option, people can feel regret [48]. More importantly, if people know in advance that they can compare outcomes, research shows that we anticipate regret and make regret-avoiding decisions [33]. In this study, we aim to link this emotion to people’s own goals. This way, anticipated regret of missing out on ones prize is meant to serve as a commitment device [47].

## Data collection

### Primary outcome

We use the Russell Standard (RS) to evaluate the outcome of the trial [49]. The primary outcome is the proportion of continuous abstinent participants 52 weeks after the initial quit date (t1 – t4). In the RS, abstinence is defined as a self-report of smoking not more than five cigarettes from the start of the abstinence period, supported by a negative biochemical validation at the final follow-up [49]. In our trial, this refers to smoking no more than five cigarettes between the quit day (t1) and the 52-week follow-up (t4). Smoking abstinence will be assessed via self-reports and is validated biochemically.

### Smoking abstinence self-reports

In both treatments, participants receive weekly text-messages from the start of the training until 13 weeks after the quit-date, to ask if they did or did not smoke that week. This 7-day point prevalence is determined by asking: have you smoked at all in the past 7 days? Participants can reply to the text message with a simple yes or no. The self-reports always take place 1 day before the CO-measurements. In the lottery arm, all measurements take place before the announcement of the lottery winner.

As part of the assessment of continuous abstinence at weeks 13, 26 and 52 after the quit date, we will ask: ‘Have you smoked at all since the quit date? A: No, never; B: 1–5 cigarettes; C: More than 5 cigarettes?’. Following the RS, answer A or B and a negative biochemical test are required for the participant to be classified as abstinent [49].

### Smoking abstinence carbon monoxide measurement

To biochemically verify smoking status, we use the validated non-invasive iCOquit Smokerlyzer® (Bedfont Scientific Ltd). The iCO measures CO-levels by requiring smokers to breath into the device. It is a strictly personal device that all participants receive by mail to their home address. Participants are asked to connect the device to their smartphone in order to use accompanied app. A tailored manual and instruction video was made for this trial and is sent to participants. Previous studies using smartphone CO-meters have shown personal mobile meters are suited to distinguish smokers from non-smokers and that usability is high [50, 51].

Participants receive a text message, asking them to perform and share the CO-measurement. After the measurement, results are presented in the app, that also allows participants to directly share their results with the trial staff via mail. Following the RS, we use a cut-off point of ≤9 ppm (p.p.m.) to determine smoking status. Measurements are weekly from the start of the training until 13 weeks after the quit date (of which 7 measurements are in the weeks of the training), and once at 26 and 52 weeks. Between weeks 14-26 there is one unannounced measurement. Participants know that it will ensue, but not in which week. If there is a difference between self-reported abstinence and biochemical validation or when participants do not respond, participants are assumed to have smoked. Following the RS, a failed biochemical test classifies a participant as smoking even when this is explained by the recent smoking of one to five cigarettes allowed throughout the follow-up period [49].

To summarize, when a participant reports not to have smoked more than five cigarettes between the quit-day and week 52, and this can be biochemically verified at week 52, they are considered not to have smoked. If previous measurements contradict this according to the criteria above, the participant is not considered continuously abstinent.

### Secondary outcomes

Secondary smoking outcomes are continuous - and point prevalence abstinence at 13 weeks (t2) and 26 weeks (t3) and are measured as described above. Self-efficacy (SE) of smoking abstinence at t1-t4 and motivation to quit at baseline are measured to also study the determinants of SE and motivation to quit. We assess self-efficacy of smoking abstinence with the Dutch Smoking Abstinence Self-efficacy Questionnaire (SASEQ [52]). Motivation to quit is assessed with the Treatment Self-Regulation Questionnaire (TSRQ [53]). In the lottery arm, participants are asked about their attitudes towards the lotteries (e.g., to what extent they motivated participants to join the cessation training).

### Covariates

With online questionnaires, we assess demographics; age, gender, nationality, education level and income. We also measure nicotine dependence, which is assessed with the translated Fagerström Test for Nicotine Dependence (FTND [54]). We will control for covariates. First, standard demographics. Second, history of smoking, which is assessed through pack-years [55]. To measure pack-years, we ask in the baseline questionnaire participants how much they smoke and how long they have smoked, which yields an estimate of lifetime smoking exposure. Third, the FTND (see above).

### Effect modifiers

We will explore multiple effect modifiers and gradually build up the models to determine which has the most parsimonious fit. To determine whether regret plays a role in any potential effect, regret proneness is measured via the translated Regret Scale [56]. We use the first question of the Dutch version of the SF-12 as a generic measure of health status [57]. The SF-12 is used to explore the effect of health status on relapse rates. Similar to health status, we will explore the effect of self-reported medication use (yes-no) on relapse rates. If variables moderate the effect, results are also presented separately per group. An overview of all measures is given in Table 1.

**Table 1:** Overview of measurements

Measurements	T0 baseline	T1-T2 weeks 1-13	T3 week 26	T4 week 52
Demographics	▲			
Generic health status (SF-12)	▲	▲	▲	▲
Self-efficacy of smoking abstinence (SASEQ)	▲	▲	▲	▲
Motivation to quit (TSRQ)	▲			
Pack-years	▲			
Regret Scale	▲			
Nicotine dependence (FTND)	▲			
Smoking self-reports (text message; RS)	▲	▲	▲	▲
CO-measurements (Smokerlyzer)	▲	▲	▲	▲

### Statistical methods

Descriptive analyses (mean, standard deviation, frequencies and percentages) on nationality, age, sex, Fagerström score, education, income and pack-years are used to display the baseline composition of both groups.

### Primary outcome

The analysis of the primary outcome examines the difference in continuous smoking abstinence between intervention group and control group 52 weeks after the quit date. After 52 weeks, a multi-level logistic regression analysis will be performed. The proportion of verified continuous abstinent participants is the dependent variable. The allocation group is the independent variable. Participants are the primary unit of inference and are clustered within organizations. Random intercepts are added at the organization level to account for clustering of observations within organizations. The multi-level model estimates the treatment effects after 52 weeks, while accounting for the clustered data pattern.

### Covariates

We will adjust in the analysis for Fagerström score, education level, income age and gender (cf. Haff et al., [32]). As a sensitivity analysis, we will also run the model without the covariates. Although we expect the majority of trainings to take place online due to COVID-19, some organizations may decide to offer them in person depending on the development of the current pandemic. The dichotomous variable online versus offline will therefore also be explored as covariate and treated similarly as above. Data will be analyzed according to the intention-to-treat principle.

### Secondary outcomes

Secondary outcomes for cessation after 13 and 26 weeks will be analyzed similar to the main model. Demographics, nicotine dependence, pack-years and regret proneness will be added as an interaction term with the allocation group to the main model to investigate effect modification. Using survival analyses, we will additionally use the SF-12 measure to explore if perceived health status influences relapse. Analyses are similar for medication use. The SASEQ scores are used to explore if self-efficacy is influenced by treatment (c.f. Van den Brand et al., [58]). We will also explore the determinants of self-efficacy of smoking at baseline with standard regression. Explorations for motivation to quit are similar. To further explore if regret proneness influences treatment effects, we will also perform a regression analysis within the lottery-arm, with abstinence at 13, 26 and 52 weeks as dependent variable and regret proneness, as well as covariates from the main model as independent variable.

### Data management

Data will be stored for 10 years in secured project folders, which are only accessible to the study staff. Personal information is stored separate from study data after the trial. After the trial, the key will be stored in a secure and separate folder. All data remains solely on the RIVM servers, which are regularly backed up. Data will be gathered and processed according the GDPR.

### Time frame

The recruitment, inclusion, randomization of participants started late in 2019 and continued in the first months of 2020. However due to the COVID-19 pandemic, nearly all recruitment and inclusion was postponed until 2021. Recruitment is still active, with the last possibility to enroll in September 2022. This means that the majority of participants has started in 2021 and the last participants will be followed up to 52 weeks after the quit date in 2023.

## Discussion

This paper describes the design and protocol of the Smoke-Free Lottery, a cluster randomized trial evaluating whether lottery deadlines at the workplace will increase the effectiveness of cessation group training by increasing the number of successfully quitted smokers. It is hypothesized that the lotteries will increase abstinence rates over and above a smoking-cessation training program.

Previous studies have used traditional incentives [20] and behavioral economically designed incentives [22] for smoking cessation and also similar lotteries targeted at different health behaviors [27, 28, 31, 59]. The present trial combines lessons from previous interventions and applies them in a unique combination of intervention (lottery deadlines), setting (the workplace) and target behavior (smoking) against relatively low costs. Their additional expenses are approximately €2.50 per participant per week ((€1050/8 participants)/52 weeks). That is 0.6% of the Dutch minimum week-wage (€405.30).

By additionally measuring psychological variables with the questionnaires, we aim to parallelly gain more knowledge about the mechanisms behind the results. Our findings may contribute to identifying behavioral economic incentives aimed at supporting smoking cessation, and possibly broader health behaviors with an addictive or intertemporal character.

### Novel lottery aspects

Previous studies have offered lotteries conditional on abstinence, but with inconclusive or disappointing results [60, 61]. However, a key difference between the present trial and previous studies is that in earlier studies, the lottery incentive took a conventional transactional form. In these applications, abstinence merely meant that a person’s ticket would enter the drawing. The lottery ticket was the reward. In essence, these lotteries therefore took the form of an uncertain pay for performance scheme with lower expected value than fixed incentives, which might help explain their lower rates of success in comparison to certain rewards [20].

In the traditional lottery studies, participants never find out what would have happened if their ticket had entered the drawing. People cannot compare their current situation to what would have happened if they had made a different decision. Understandably, they know for sure that they win nothing if they smoke, but never the alternative reality. In the present trial, it is certain for participants that their ticket will enter the drawing and that they can compare outcomes. This comparison can turn out either good or bad, but there is always the risk of finding out you have won, but that your own behavior has resulted in having to give up the prize. The financial outcome may be identical as in a traditional lottery (smoking means no prize), but the anticipated emotion and intensity are expected to differ [48]. In national lotteries, the promise of this counterfactual feedback has motivated people to prevent regret and play, more often than in traditional lotteries where if you not decide to play, you never find out what would have happened if you had played [47]. This present trial can help answer if this design can overcome the shortcomings of previous lottery interventions for smoking cessation.

### Lottery schedule

Abstinence in the early stages of quitting is the most important predictor of long-term success [41]. Therefore, we offer the weekly lottery-deadlines immediately after the quit-date. To further prevent unsuccessful participants from exiting the program, we offer all incentives, regardless of prior success. If a participant smoked, they can still participate in the upcoming lottery. This provides the opportunity to start over after a relapse and is designed to alleviate a possible all or nothing feeling of disappointment. Despite high numbers of adherence, a substantial proportion of relapse has also been observed in the 3 months after the group training that is offered in this study [21]. In general, lower-SES smokers also tend to drop out of cessation services earlier [9]. Research into the shape of the relapse curve further shows that after 26 weeks, the probability of relapse is relatively stable in comparison to earlier [38–41]. As a result, it might be beneficial to provide a long-term maintenance deadline until 26 weeks on top of initial repeated short-term deadlines [62]. The present trial builds on this reasoning and can show if this fits smokers’ needs for support.

The current schedule and rules have a limitation that is worth mentioning. As the prize at week 26 is drawn until a winner is awarded, there is for an individual participant, from a purely financial point of view, a monetary benefit if their fellow group members relapse into smoking as this increases their chances of winning. This is an unintended design feature that should be addressed in future applications of similar lotteries.

### Existing motivation and co-workers

Regularly accompanied with the offering of interventions with a financial component, there is the concern that this will negatively affect participants’ intrinsic motivation or confidence in their own ability for attaining the goal [63, 64]. However, a review of the literature finds no evidence of crowding out by incentives in the domain of health behavior [64]. There is also evidence of positive motivational effects of financial incentives; immediate rewards can increase intrinsic motivation [65], and self-efficacy has been found to mediate the beneficial effect of financial incentives on smoking cessation [58]. Data from the present trial could further enlighten these effects, applied to lotteries.

Another concern is that non-smoking employees might envy their smoking co-workers because of the prizes. Qualitative research shows that in practice, most employees support their colleagues out of solidarity, acknowledge that quitting is difficult and do not resent them for receiving financial support [66].

### Strengths and limitations

The current trial is subject to several limitations. First, randomizing clusters and not participants increases the probability that intracluster effects influence results. That is, observations within clusters are correlated. As a result, the required sample size is higher than would be the case with randomization at the participant level and clustering must be accounted for in the statistical models. We aim to account for clustering in the multilevel model by allowing random intercepts.

Randomization at the cluster level has the practical benefit that employers can communicate clearly what the program entails to the entire company at once. A company’s limited communication-resources (mostly time) and competing internal messaging (e.g., newsletters) mean that raising employees’ attention for participation in scientific studies requires highly digestible homogeneous information. Our experience is that one key message, communicated to a single cluster improves this.

A methodological benefit of cluster randomization is that it can avoid treatment contamination of participants within companies or groups [67]. Contamination occurs when participants in the control arm receive active intervention influences from the intervention arm. This makes the control arm more similar to the intervention arm, reduces their intended randomized contrast and hinders the possibly for causal inference [67].

In our trial, randomization at the individual level would have resulted in participants in one training group receiving different treatments. In that case, cessation group members allocated to the control arm could be influenced positively by their peers receiving incentives or negatively as they are not eligible for a prize, while their peers are. Likewise, participants in the intervention arm interacting with nearby participants in the control arm might share their prizes with those not allocated to the lotteries. Taken together, cluster randomization was reasoned to be the best design for testing a group-based intervention in this context.

Another limitation of first randomizing employers and next starting the recruitment of employees, is that the training + lotteries arm might especially attract smokers who are interested in the lotteries (see *Recruitment).* However, allocating participants after enrollment does not fully rule out this motivation, as participants might still participate hoping to be randomized to the lottery arm, possibly enhancing disappointment after. The benefit of our approach is that we minimize demotivation and maximize clarity at the employee level early on. For example, several employers stated that they would only participate if they would be enrolled to the lottery arm, therefore did not meet our eligibility criteria and could not participate in the study. In the surveys, we attempt to assess motivation to participate among employees.

We require organizations to pay for the training and lotteries and require participants to own a smartphone. Therefore, a third limitation is that our recruitment strategy might favor relatively wealthier organizations and employees. This risk is minimized by the fact that the training is covered by employees’ health insurance (see *Setting)* and that the total lottery expenses are kept low for employers (0.6% of the Dutch minimum week-wage). Yet, if the tested intervention is successful and subject to further scaling, it could be considered to a) fully fund the lotteries and b) provide tailored communication for participants without a smartphone in order to minimize inequality in reach and uptake. A fourth limitation is the process of biochemical verification of self-reports. The primary outcome relies on participants willingness to measure and submit their CO-values long after the training has ended. We aim to realize this by offering a time window (c.f., the RS), our retention strategy and by sending reminders. In addition, CO-measurements are an accepted and widely used method [49], but cannot guarantee abstinence over the full 52 weeks. We assess continuous abstinence with two instruments at multiple points in time and include a surprise measurement, but cannot rule out that a negative CO-measurement is the result of only recent smoking cessation.

An adjacent limitation is that unsuccessful participants might game the measurement by, for example, asking a non-smoker in their environment to breath into the device. In previous instances, CO-measurements were compared with urinary and salivary cotinine and only 4% of smokers reported falsely [68]. Likewise, even when there was a significant financial benefit in cheating, a trial with 604 participants found no differences between self-reports and CO-measurements [21]. We aim to prevent cheating by first asking to self-report smoking status and requiring the CO-measurement a day later and by stressing to participants that either smoking status (smoker or non-smoker) is acceptable to receive the study payment at the end of the study. At the main outcome point, there is also no financial incentive for cheating in either arm. By also using trusting and supporting langue throughout the trial (e.g., stating that relapse is never a personal failure), we aim to minimize this risk further.

A final limitation of the current study design is that it does not allow to disentangle the psychological mechanisms responsible for a potential effect. The lotteries host multiple components to leverage well-known influences on decision-making [26]. While we use surveys to explore perceptions and psychological constructs at work, trials with more than two treatment arms allow to vary more design characteristics to identify working mechanisms more precisely [69].

An important benefit of the current study design is the 6-month follow-up, after all lotteries have ended. A common pattern in the application of the current lotteries is that they especially support initial health behavior change, which declines after removal of the lottery deadlines [31, 32]. In contrast, research into smoking relapse curves suggest that not smoking might become ‘easier’ over time [41]. The present design allows us to explore which of the two patterns will be dominant.

### Practical implications

Results of this trial can be used to improve cessation programs at the workplace. While it is known that incentives can work [20], and that the workplace is a suitable intervention-context [8], employers can hesitate because of opportunity costs, fairness and questions about effectiveness [66]. This study can further answer if relatively low-cost lotteries can also improve cessation. For this trial, we require employers to pay for the training and the lotteries, which resembles the situation if this method shows to be effective and is implemented in practice. If the Smoke-free Lottery is effective, it could be studied if it works in broader contexts and how its implementation could be facilitated to support the many smokers that want to quit, but could use some form of commitment in realizing their own goal.

### Conclusion

This paper presents the design and protocol of a cluster randomized trial to evaluate a smoking cessation intervention paired with lottery deadlines at the workplace. The results of this study could provide insights into the effectiveness of the incentives in combination with smoking cessation program at the workplace and several underlying psychological mechanisms. If effective, the lotteries could be a relatively low-cost addition to existing support.

## Supplementary Information

**Additional file 1.**

## Data Availability

The datasets used and/or analyzed during the current study will be available from the corresponding author upon reasonable request.

## Abbreviations

CO: Carbon monoxide
Pack-year: Number of daily packs x years
CRT: Cluster randomized trial
ID: Identifier
RS: Russell Standard
SE: Self efficacy
SASEQ: Smoking Abstinence Self-efficacy Questionnaire
TSRQ: Treatment Self-Regulation Questionnaire
FTND: Fagerström Test for Nicotine Dependence
SF12: Short Form Health Survey 12
GDPR: General Data Protection Regulation
SES: Socioeconomic status

## References

[1] Murray CJL, Aravkin AY, Zheng P, Abbafati C, Abbas KM, Abbasi-Kangevari M (2020). Global burden of 87 risk factors in 204 countries and territories, 1990–2019: a systematic analysis for the global burden of disease study 2019. Lancet.

[2] Gregoraci G, van Lenthe FJ, Artnik B, Bopp M, Deboosere P, Kovács K (2017). Contribution of smoking to socioeconomic inequalities in mortality: a study of 14 European countries, 1990-2004. Tob Control.

[3] Jha P, Peto R (2014). Global effects of smoking, of quitting, and of taxing tobacco. N Engl J Med.

[4] Goodchild M, Nargis N, d'Espaignet ET (2018). Global economic cost of smoking-attributable diseases. Tob Control.

[5] Troelstra SA, Coenen P, Boot CR, Harting J, Kunst AE, van der Beek AJ (2020). Smoking and sickness absence: a systematic review and meta-analysis. Scand J Work Environ Health.

[6] Weng SF, Ali S, Leonardi-Bee J (2013). Smoking and absence from work: systematic review and meta-analysis of occupational studies. Addiction.

[7] Berman M, Crane R, Seiber E, Munur M (2014). Estimating the cost of a smoking employee. Tob Control.

[8] Cahill K, Lancaster T. Workplace interventions for smoking cessation. Cochrane Database Syst Rev. 2014;(2):1-105. 10.1002/14651858.CD003440.pub4 PMC11285308 24570145

[9] Brown T, Platt S, Amos A (2014). Equity impact of European individual-level smoking cessation interventions to reduce smoking in adults: a systematic review. Eur J Pub Health.

[10] Brown T, Platt S, Amos A (2014). Equity impact of population-level interventions and policies to reduce smoking in adults: a systematic review. Drug Alcohol Depend.

[11] Nagelhout GE, Willemsen MC, de Vries H (2011). The population impact of smoke-free workplace and hospitality industry legislation on smoking behaviour. Findings from a national population survey. Addiction.

[12] Kock L, Brown J, Hiscock R, Tattan-Birch H, Smith C, Shahab L (2019). Individual-level behavioural smoking cessation interventions tailored for disadvantaged socioeconomic position: a systematic review and meta-regression. Lancet Public Health.

[13] Chaiton M, Diemert L, Cohen JE, Bondy SJ, Selby P, Philipneri A (2016). Estimating the number of quit attempts it takes to quit smoking successfully in a longitudinal cohort of smokers. BMJ Open.

[14] Bickel WK, Odum AL, Madden GJ (1999). Impulsivity and cigarette smoking: delay discounting in current, never, and ex-smokers. Psychopharmacology.

[15] Bradford D, Courtemanche C, Heutel G, McAlvanah P, Ruhm C (2017). Time preferences and consumer behavior. J Risk Uncertain.

[16] Wang Y, Sloan FA (2018). Present bias and health. J Risk Uncertain.

[17] Loewenstein G (2005). Hot-cold empathy gaps and medical decision making. Health Psychol.

[18] Hagger MS, Cameron LD, Hamilton K, Hankonen N, Lintunen T (2020). The handbook of behavior change.

[19] Gneezy U, Kajackaite A, Meier S. Incentive-Based Interventions. In: Hagger MS, Cameron LD, Hamilton K, Hankonen N, Lintunen T, editors. The Handbook of Behavior Change. New York: Cambridge University Press; 2020. p. 523-536.

[20] Notley C, Gentry S, Livingstone-Banks J, Bauld L, Perera R, Hartmann-Boyce J. Incentives for smoking cessation. Cochrane Database Syst Rev. 2019;(7):1-120. 10.1002/14651858.CD004307.pub6 PMC6635501 31313293

[21] van den Brand FA, Nagelhout GE, Winkens B, Chavannes NH, van Schayck OCP (2018). Effect of a workplace-based group training programme combined with financial incentives on smoking cessation: a cluster-randomised controlled trial. Lancet Public Health.

[22] Volpp KG, Troxel AB, Pauly MV, Glick HA, Puig A, Asch DA (2009). A randomized, controlled trial of financial incentives for smoking cessation. N Engl J Med.

[23] Adams J, Giles EL, McColl E, Sniehotta FF (2014). Carrots, sticks and health behaviours: a framework for documenting the complexity of financial incentive interventions to change health behaviours. Health Psychol Rev.

[24] Struijs J, Hayen A, Van der Swaluw K. Health Affairs Blog 2018. 10.1377/hblog20180420.640240/full/.

[25] Breen RJ, Ferguson SG, Palmer MA (2020). Higher incentive amounts do not appear to be associated with greater quit rates in financial incentive programmes for smoking cessation. Addict Behav.

[26] Vlaev I, King D, Darzi A, Dolan P (2019). Changing health behaviors using financial incentives: a review from behavioral economics. BMC Public Health.

[27] Volpp KG, John LK, Troxel AB, Norton L, Fassbender J, Loewenstein G (2008). Financial incentive–based approaches for weight loss: a randomized trial. JAMA.

[28] Kimmel SE, Troxel AB, Loewenstein G, Brensinger CM, Jaskowiak J, Doshi JA (2012). Randomized trial of lottery-based incentives to improve warfarin adherence. Am Heart J.

[29] Patel MS, Asch DA, Rosin R, Small DS, Bellamy SL, Eberbach K, et al. Individual versus team-based financial incentives to increase physical activity: a randomized, controlled trial. J Gen Intern Med. 2016;31:746-754. 10.1007/s11606-016-3627-0 PMC4907949 26976287

[30] van der Swaluw K, Lambooij MS, Mathijssen JJP, Schipper M, Zeelenberg M, Berkhout S (2018). Commitment lotteries promote physical activity among overweight adults—a cluster randomized trial. Ann Behav Med.

[31] van der Swaluw K, Lambooij MS, Mathijssen JJP, Schipper M, Zeelenberg M, Berkhout S (2018). Physical activity after commitment lotteries: examining long-term results in a cluster randomized trial. J Behav Med.

[32] Haff N, Patel MS, Lim R, Zhu J, Troxel AB, Asch DA (2015). The role of behavioral economic incentive design and demographic characteristics in financial incentive-based approaches to changing health behaviors: a meta-analysis. Am J Health Promot.

[33] Zeelenberg M (1999). Anticipated regret, expected feedback and behavioral decision making. J Behav Decis Mak.

[34] Gezondheidsenquête/Leefstijlmonitor. CBS & RIVM. 2021.

[35] Schulz KF, Grimes DA (2002). Generation of allocation sequences in randomised trials: chance, not choice. Lancet.

[36] Hajek P (1989). Withdrawal-oriented therapy for smokers. Br J Addict.

[37] Miller WR, Rollnick S (2012). Meeting in the middle: motivational interviewing and self-determination theory. Int J Behav Nutr Phys Act.

[38] Hughes JR, Keely J, Naud S (2004). Shape of the relapse curve and long-term abstinence among untreated smokers. Addiction.

[39] Garvey AJ, Bliss RE, Hitchcock JL, Heinold JW, Rosner B (1992). Predictors of smoking relapse among self-quitters: a report from the normative aging study. Addict Behav.

[40] Jackson SE, McGowan JA, Ubhi HK, Proudfoot H, Shahab L, Brown J (2019). Modelling continuous abstinence rates over time from clinical trials of pharmacological interventions for smoking cessation. Addiction.

[41] Kirshenbaum AP, Olsen DM, Bickel WK (2009). A quantitative review of the ubiquitous relapse curve. J Subst Abus Treat.

[42] Rogers T, Milkman KL, Volpp KG (2014). Commitment devices: using initiatives to change behavior. JAMA.

[43] Thaler RH, Shefrin HM (1981). An economic theory of self-control. J Polit Econ.

[44] Ariely D, Wertenbroch K (2002). Procrastination, deadlines, and performance: self-control by precommitment. Psychol Sci.

[45] Kahneman D, Tversky A. Prospect theory: an analysis of decision under risk. Econometrica. 1979;47(2):263–91.

[46] Loewenstein GF, Weber EU, Hsee CK, Welch N (2001). Risk as feelings. Psychol Bull.

[47] Zeelenberg M, Pieters R (2004). Consequences of regret aversion in real life: the case of the Dutch postcode lottery. Organ Behav Hum Decis Process.

[48] Van der Swaluw K, Lambooij MS, Mathijssen JJP, Zeelenberg M, Polder JJ, Prast HM (2018). Emotional responses to behavioral economic incentives for health behavior change. Psychology Health Med.

[49] West R, Hajek P, Stead L, Stapleton JJA (2005). Outcome criteria in smoking cessation trials: proposal for a common standard. Addiction.

[50] Meredith SE, Robinson A, Erb P, Spieler CA, Klugman N, Dutta P (2014). A mobile-phone-based breath carbon monoxide meter to detect cigarette smoking. Nicotine Tob Res.

[51] Beard E, West R (2012). Pilot study of the use of personal carbon monoxide monitoring to achieve radical smoking reduction. J Smok Cessat.

[52] Spek V, Lemmens F, Chatrou M, van Kempen S, Pouwer F, Pop V (2013). Development of a smoking abstinence self-efficacy questionnaire. Int J Behav Med.

[53] Levesque CS, Williams GC, Elliot D, Pickering MA, Bodenhamer B, Finley PJ (2006). Validating the theoretical structure of the treatment self-regulation questionnaire (TSRQ) across three different health behaviors. Health Educ Res.

[54] Kozlowski LT, Porter CQ, Orleans CT, Pope MA, Heatherton T (1994). Predicting smoking cessation with self-reported measures of nicotine dependence: FTQ, FTND, and HSI. Drug Alcohol Depend.

[55] Bernaards CM, Twisk JW, Snel J, Van Mechelen W, Kemper HC (2001). Is calculating pack-years retrospectively a valid method to estimate life-time tobacco smoking? A comparison between prospectively calculated pack-years and retrospectively calculated pack-years. Addiction.

[56] Schwartz B, Ward A, Monterosso J, Lyubomirsky S, White K, Lehman DR (2002). Maximizing versus satisficing: happiness is a matter of choice. J Pers Soc Psychol.

[57] Ware JE, Kosinski M, Keller SD (1996). A 12-item short-form health survey: construction of scales and preliminary tests of reliability and validity. Med Care.

[58] van den Brand FA, Candel MJJM, Nagelhout GE, Winkens B, van Schayck CP (2020). How financial incentives increase smoking cessation: a two-level path analysis. Nicotine Tob Res.

[59] Haisley E, Volpp KG, Pellathy T, Loewenstein G (2012). The impact of alternative incentive schemes on completion of health risk assessments. Am J Health Promot.

[60] Cahill K, Perera R. Quit and win contests for smoking cessation. Cochrane Database Syst Rev. 2008;(4):1-28. 10.1002/14651858.CD004986.pub3 18843674

[61] Fanshawe TR, Hartmann-Boyce J, Perera R, Lindson N. Competitions for smoking cessation. Cochrane Database Syst Rev. 2019;(2):1-56. 10.1002/14651858.CD013272 PMC6953205 30784046

[62] Van der Swaluw K, Lambooij MS, Mathijssen JJP, Schipper M, Zeelenberg M, Polder JJ (2016). Design and protocol of the weight loss lottery- a cluster randomized trial. Contemp Clin Trials.

[63] Deci EL, Koestner R, Ryan RM (1999). A meta-analytic review of experiments examining the effects of extrinsic rewards on intrinsic motivation. Psychol Bull.

[64] Promberger M, Marteau TM (2013). When do financial incentives reduce intrinsic motivation? Comparing behaviors studied in psychological and economic literatures. Health Psychol.

[65] Woolley K, Fishbach A (2018). It’s about time: earlier rewards increase intrinsic motivation. J Pers Soc Psychol.

[66] van den Brand FA, Magnée T, de Haan-Bouma L, Barendregt C, Chavannes NH, van Schayck OCP (2019). Implementation of financial incentives for successful smoking cessation in real-life company settings: a qualitative needs assessment among employers. Int J Environ Res Public Health.

[67] Magill N, Knight R, McCrone P, Ismail K, Landau S (2019). A scoping review of the problems and solutions associated with contamination in trials of complex interventions in mental health. BMC Med Res Methodol.

[68] Ierfino D, Mantzari E, Hirst J, Jones T, Aveyard P, Marteau TM (2015). Financial incentives for smoking cessation in pregnancy: a single-arm intervention study assessing cessation and gaming. Addiction.

[69] Milkman KL, Gromet D, Ho H, Kay JS, Lee TW, Pandiloski P (2021). Megastudies improve the impact of applied behavioural science. Nature.

